# Identification, mapping, and self-reported practice patterns of village doctors in Sitakunda subdistrict, Bangladesh

**DOI:** 10.7189/jogh.14.04185

**Published:** 2024-09-13

**Authors:** Olivia R Hanson, Ishtiakul I Khan, Zahid Hasan Khan, Mohammad Ashraful Amin, Debashish Biswas, Md Taufiqul Islam, Eric J Nelson, Sharia M Ahmed, Ben J Brintz, Sonia T Hegde, Firdausi Qadri, Melissa H Watt, Daniel T Leung, Ashraful I Khan

**Affiliations:** 1 Division of Infectious Diseases, Department of Internal Medicine, Spencer Fox Eccles School of Medicine at the University of Utah, Salt Lake City, Utah, USA; 2 Infectious Diseases Division, International Center for Diarrhoeal Disease Research, Bangladesh, Dhaka, Bangladesh; 3 School of Population and Global Health, The University of Western Australia, Perth, Australia; 4 Health System and Population Studies Division, International Center for Diarrhoeal Disease Research, Bangladesh, Dhaka, Bangladesh; 5 Departments of Pediatrics and Environmental and Global Health, University of Florida, Gainesville, Florida, USA; 6 Division of Epidemiology, Department of Internal Medicine, Spencer Fox Eccles School of Medicine at the University of Utah, Salt Lake City, Utah, USA; 7 Department of Epidemiology, Johns Hopkins Bloomberg School of Public Health, Baltimore, Maryland, USA; 8 Department of Population Health Sciences, Spencer Fox Eccles School of Medicine at the University of Utah, Salt Lake City, Utah, USA

## Abstract

**Background:**

Informally trained health care providers, such as village doctors in Bangladesh, are crucial in providing health care services to the rural poor in low- and middle-income countries. Despite being one of the primary vendors of antibiotics in rural Bangladesh, village doctors often have limited knowledge about appropriate antibiotic use, leading to varied and potentially inappropriate dispensing and treatment practices. In this study, we aimed to identify, map, and survey village doctors in the Sitakunda subdistrict of Bangladesh to understand their distribution, practice characteristics, clinical behaviours, access to technologies, and use of these technologies for clinical decision-making.

**Methods:**

Using a ‘snowball’ sampling method, we identified and mapped 411 village doctors, with 371 agreeing to complete a structured survey.

**Results:**

The median distance between a residential household and the closest village doctor practice was 0.37 km, and over half of the practices (51.2%) were within 100 m of the major highway. Village doctors were predominately male (98.7%), with a median age of 39. After completing village doctor training, 39.4% had completed an internship, with a median of 15 years of practice experience. Village doctors reported seeing a median of 84 patients per week, including a median of five paediatric diarrhoea cases per week. They stocked a range of antibiotics, with ciprofloxacin and metronidazole being the most prescribed for diarrhoea. Most had access to phones with an internet connection and used online resources for clinical decision-making and guidance.

**Conclusions:**

The findings provide insights into the characteristics and practices of village doctors and point to the potential for internet and phone-based interventions to improve patient care and reduce inappropriate antibiotic use in this health care provider group.

The provision of health care services in low- and middle-income countries (LMICs) heavily relies on informally trained health care providers, who are responsible for up to 90% of health care interactions in these regions [1]. Bangladesh is particularly reliant on informally trained providers, given the shortage of qualified health care professionals in rural areas [2]. The most common among them are village doctors, who practice allopathic medicine without formal medical training, dispense medications, and treat basic medical conditions. These doctors play a crucial role as frontline health care providers, especially in rural areas, and comprise a significant proportion of the health care system in Bangladesh [3]. Data suggest that village doctors and other informal providers serve as the first line of care for 65% of patients and the sole source of care for 46% of patients in rural areas of Bangladesh [4]. Village doctors establish private practices that are easily accessible, with storefronts strategically located in high-traffic areas, and operate during the daytime as well as evenings and weekends when other public outpatient facilities are usually unavailable [5]. Despite their pivotal role, village doctors generally have limited formal education [6,7], with most acquiring short-term training on common illnesses from unregulated private institutions [7,8].

Across LMICs, informal health care providers, including village doctors, traditional healers, and community health workers, form integral components of health care delivery in rural areas with limited access to formal health care [9–11]. Their close ties to the community enable them to closely engage with local populations, improving health care utilisation and access in rural areas, and bridging the gap between rural communities and formal health care systems [12–14]. These informal care providers play a key role in Bangladesh’s pluralistic health system, which blends centrally planned government-controlled public health services with informal care providers [15]. The government's efforts have supported health care infrastructure and essential services, contributing to improved health outcomes [15]. Simultaneously, informal providers such as village doctors have complemented formal health care, providing more affordable and accessible care while leveraging community ties [8]. In Bangladesh, village doctors are one of the primary sources of antibiotics [16]. However, these providers’ limited knowledge about appropriate antibiotic use has led to varied and broad antibiotic dispensing practices, resulting in unintended harmful consequences, including unnecessary side effects at the patient level and contributions to antimicrobial-resistant pathogens at the population level [2].

Studies have documented patterns of antibiotic resistance in Bangladesh, with alarming rates of increase in resistance rates for commonly used antibiotics such as ampicillin and ciprofloxacin [17]. These findings reflect broader global trends, with LMICs being disproportionately affected due to the higher burden of communicable diseases [18]. Notably, the misuse of antibiotics contributes to the persistence and dissemination of resistant strains, posing significant challenges to infection control and treatment efficacy [17,19]. Furthermore, the overuse and inappropriate prescribing of antibiotics has been linked to adverse health outcomes, including increased incidence of treatment failures, prolonged illness duration, and higher health care costs [20,21]. Combating antibiotic resistance is a central focus of Bangladesh's national health policies, particularly demonstrated by the National Action Plan on Antimicrobial Resistance launched in 2017, which places an emphasis on surveillance enhancement, appropriate antibiotic use promotion, infection prevention and control strengthening, and improving antimicrobial stewardship in health care settings [22]. In alignment with national public health priorities, addressing the issue of inappropriate antibiotic use among informal health care providers is imperative to reduce harmful health effects and stop the rise of antibiotic resistance in Bangladesh. Given the prominent role of village doctors, interventions to reduce the misuse of antibiotics should specifically target this group of health care providers.

Antibiotics for diarrhoeal diseases account for a high proportion of antibiotic utilisation by village doctors. In a study in peri-urban Bangladesh, nearly 100% of encounters between village doctors and patients with diarrhoeal illness resulted in antibiotics being prescribed or dispensed, in contrast to 30% of encounters with community health workers [2]. Higher rates of antibiotic dispensing by village doctors may be driven by financial incentives and worsened by their inconsistent or limited knowledge about antibiotics [6].

Insights into the attitudes and practices of village doctors can inform practical interventions to promote appropriate antibiotic utilisation and stewardship in the community. Moreover, this understanding plays a pivotal role in the development of targeted training programmes and regulatory measures tailored to the needs of informal health care providers. By pinpointing specific knowledge gaps and aligning regulatory frameworks with common practices, such interventions can effectively enhance health care practices and outcomes, addressing the unique challenges faced by village doctors and their communities. With this study, our objective was to locate and map all village doctors providing health care services in the Sitakunda Upazila subdistrit of Bangladesh and describe their characteristics and clinical practices concerning the treatment of diarrhoeal illnesses. Furthermore, we aimed to describe village doctors’ utilisation of Internet and phone-based resources to determine the possibility of implementing an electronic clinical decision support tool (eCDST) to assist health care providers in effectively managing patients and promoting responsible antibiotic use.

## METHODS

This survey was part of a larger study aimed at assessing the feasibility of implementing an eCDST in village doctor practices, specifically to reduce the inappropriate use of antibiotics. Because comprehensive records of village doctor locations and practice characteristics were unavailable in the Sitakunda Upazila, a primary survey was necessary to describe the village doctor practices in the region. Between February and April 2023, we aimed to identify all village doctors in Sitakunda Upazila to record their location coordinates for mapping and collect data on their characteristics and practices through a questionnaire. All study procedures were approved by the institutional review committees (Research Review Committee and Ethical Review Committee) of the International Center for Diarrhoeal Disease Research, Bangladesh (icddr,b) and the University of Utah.

### Setting

**Figure 1:**
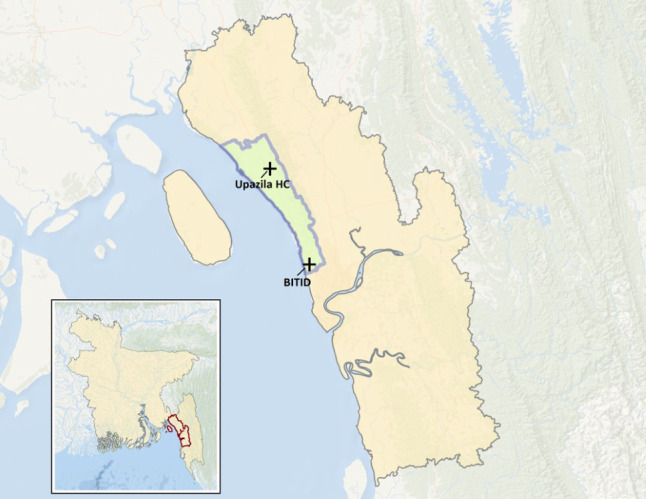
Map of Chattogram district showing the Sitakunda Upazila (subdistrict) outlined in blue. The + symbols indicate the location of the Sitakunda Upazila Health Complex and BITID Hospital. The inset map shows the area of Chattogram district within Bangladesh.

We conducted this study in Southeastern Bangladesh in the Sitakunda Upazila, located within the Chattogram District, Chattogram Division (**Figure 1**). Sitakunda Upazila encompasses an approximate area of 500 km^2^, with a population of around 383 000 individuals [23]. It is bordered by the Sandwip Channel of the Bay of Bengal to the west and neighbouring subdistricts, including Mirsharai to the north, Pahartali to the south, and Fatickchhari and Hathazari to the east. Chattogram, the second largest city in Bangladesh, with a metropolitan population of 5.38 million as of 2023, borders Sitakunda to the south [24]. Sitakunda comprises ten unions, the lowest administrative unit, each with a population of 20 000 to 55 000, and includes one designated urban settlement known as Sitakunda Town, which has a population of 36 650.

The ten unions of Sitakunda Upazila collectively house 112 villages, according to the 2011 Bangladesh census [25]. Within Sitakunda, there are several health care facilities, including the Sitakunda Upazila Health Complex, a subdistrict health facility, and the Bangladesh Institute of Tropical Infectious Diseases (BITID), a tertiary-care referral hospital. The research team selected Sitakunda as the location of the study, given its accessibility, existing infrastructure, and the team’s previous work in the area and longstanding relationship with BITID.

### Procedures

In this study, a village doctor refers to any informal health care provider or drug vendor who practices allopathic medicine without formal training. Most village doctors, locally known as *gram dakter*, provide patient care, dispense medications, and operate medicine shops. To be eligible, participants needed to be drug vendors or informal providers who see patients in a chamber practice and prescribe or dispense medications.

Data collection and fieldwork were carried out between February and April 2023 by surveyors based at BITID, who have extensive familiarity with the local population. icddr,b investigators based at BITID had previously established relationships with village doctors throughout Sitakunda. Surveyors initially reached out to village doctors with whom they already knew and identified additional village doctors using a snowball sampling method. They then obtained verbal consent, recorded the latitude and longitude coordinates of village doctors’ practice locations (regardless of whether the village doctor consented to complete the survey) and afterwards administered a 28-question survey. The survey was conducted in a private space to ensure confidentiality, and participants were allowed to ask questions for clarification.

The icddr,b research team designed the survey, developing questions around nine areas of focus: type of practice, staffing, technology, finances, education, oversight, scope of practice, relationships, and geospatial. They pre-tested the survey instrument before implementation by administering it to a small sample of village doctors in Sitakunda to assess the clarity of questions, understand the feasibility of data collection methods, and identify any potential issues with survey administration. The survey questionnaire was refined through feedback from field visits and discussions with village doctors. The survey instrument was orally administered to the participants in Bangla, and the research staff member recorded responses on a paper form. The survey included questions about demographics; training and education history; practice characteristics; patient population and load; medication and antibiotic usage; disease management practices; and referral practices. When inquiring about antibiotic stocking and dispensing practices, the survey asked about antibiotics commonly prescribed for diarrhoeal disease in Bangladesh, including metronidazole, azithromycin, doxycycline, ciprofloxacin, nitazoxanide, and ceftriaxone. Additionally, the survey asked about access to smartphones and the internet, the current use of these technologies for clinical decision-making, and other relevant information to determine the potential for introducing an eCDST. The full questionnaire is available in the **Online Supplementary Document**. Throughout the data collection process, the research team had regular debriefing meetings to ensure consistency and quality in both sampling and data collection procedures.

### Data management and analysis

We plotted the location coordinates of the village doctor practices onto a digitised map of the Sitakunda Upazila using ArcGIS, version 10.8.2 (Esri, Redlands, California, USA). Using a previously published approach for counting households in Sitakunda, we used satellite imagery (Airbus, Pléiades 1B Sensor) with digitised building footprints to classify single or multi-story units [26–28]. To validate these data, we conducted a dwelling assessment by visiting a sample of satellite-identified areas in Sitakunda to determine how many were occupied households and how many households existed within each area. After dividing the Sitakunda Upazila into 1km^2^ grid cells, the summed number of households per grid cell (assuming an average household size of four individuals) was highly correlated with the population density per grid cell, as derived from WorldPop [29]. We used haversine distance to calculate and summarise the distance from each household to the nearest village doctor.

We then entered the survey responses into an Excel spreadsheet and exported them to SPSS, version 27 (IBM Corporation, Armonk, New York, USA) for analysis. We conducted basic descriptive analyses, including proportions for categorical data and the median, interquartile range (IQR), and range for continuous variables.

## RESULTS

### Geographic distribution of village doctors

**Figure 2:**
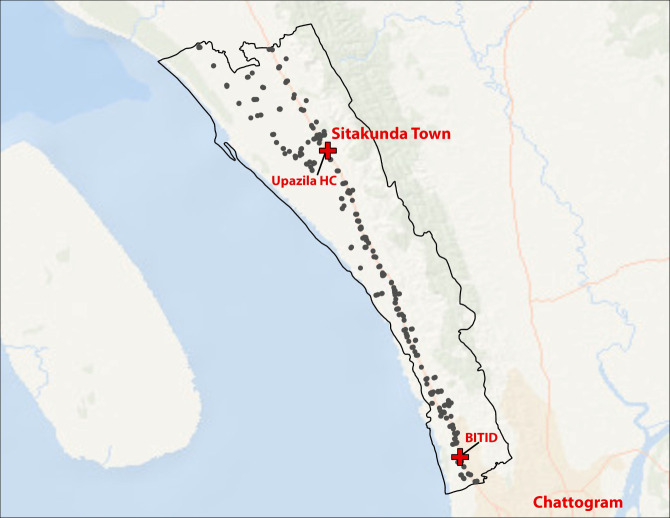
Distribution of village doctors throughout the Sitakunda Upazila. Black dots indicate the location of village doctors. Red crosses indicate the presence of Sitakunda Upazila and BITID Hospital.

We identified 411 village doctors in Sitakunda Upazila, of whom 371 (90.2%) responded to the survey, indicating at least one village doctor practice per 1000 people. Village doctors were predominantly located along or adjacent to major roadways (**Figure 2**). More than half of the village doctor practices (n = 210, 51.1%) were located within 100 m of the Dhaka-Chittagong Highway, the only major roadway running through Sitakunda. Most village doctors (n = 329, 80.0%) were located within 1 km of the Dhaka-Chittagong Highway. The density of village doctor practices was higher in Sitakunda Town and peri-urban areas around Chattogram and lower in more rural areas, particularly in the northern unions of Sitakunda. The median distance between households and a village doctor practice was 0.37 km (IQR = 0.18–0.66, range = 0.00–5.06).

### Village doctor characteristics

The village doctors had a median age of 39 years (IQR = 32–47, range = 20–74) and had been practising for a median of 15 years (IQR = 8–22, range = 1–48). Most of that time was spent practising in Sitakunda, with the median time being 14 years (IQR = 7–21, range = 1–48). The median time practising at their current location was eight years (IQR = 4–18, range = 0.16–55). Almost all participants had completed some informal or formal training related to being a village doctor, with the most common training being an eight-month-long course entitle ‘Local Medical Assistant & Family Welfare/Planning’ (n = 218, 58.8%) [30]. More than half (n = 197, 53.1%) reported training and qualifications through the Pharmacy Council Bangladesh, an autonomous organisation that offers pharmacy-related education. Over a third of participants (n = 133, 35.8%) reported completing the three-month-long ‘Rural Medical Practitioner’ training [30]. Very few village doctors (n = 20, 5.4%) reported being self-trained. Over one-third (n = 146, 39.4%) completed an internship after village doctor training, with the median internship length being three months (IQR = 1.25–6, range = 1–12). We also recorded additional demographics, including gender, religion, education, and employment status (**Table 1**).

**Table 1:** Demographics of village doctors practising in the Sitakunda Upazila (n = 371)

Variables by category	n (%)
Gender	
*Male*	366 (98.7)
*Female*	5 (1.3)
Religion	
*Hindu*	186 (50.1)
*Muslim*	185 (49.9)
Completed education levels (before village doctor training)	
*Class 6–8)*	4 (1.1)
*Secondary school certificate*	110 (29.6)
*Higher secondary school certificate (class 11–12)*	147 (39.6)
*Graduation from general studies*	92 (24.8)
*Post-graduation education*	18 (4.9)
Has another job in addition to being a village doctor	
*Yes*	46 (12.4)
*No*	325 (87.6)

Almost all village doctors rented their practice location (n = 342, 92.2%), were open seven days a week (n = 370, 99.7%), and made house calls in addition to seeing clients on site (n = 344, 92.7%). Most village doctors had a clinical supervisor (n = 254, 68.5%), and over a third (n = 139, 37.5%) had at least one employee they supervised. About half (n = 194, 52.3%) stated that they did not charge a consultation fee, while the mean price for those who did charge a fee was BDT 50, equivalent to USD 0.45 (in November 2023) (IQR = 50–100; range = 20–200). Village doctors self-reported their monthly earnings (including consultation fees, drug vending, and other revenue) as a mean of BDT 20 000, equivalent to approximately USD 180 in November 2023 (IQR = 20 000–25 000, range = 10 000–50 000).

### Clinical behaviours

**Figure 3:**
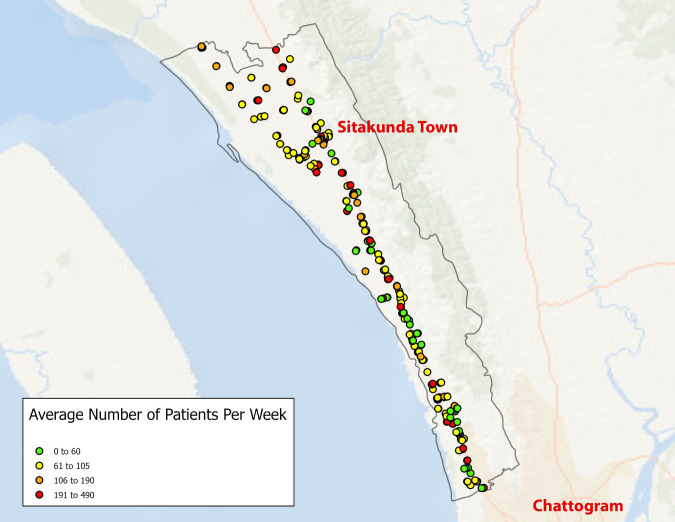
The average number of patients seen at village doctor practices in the Sitakunda Upazila per week.

Village doctors reported a median number of 84 (IQR = 70–140, range = 20–490) total patients per week, including a median of 21 (IQR = 10–35, range = 0–150) children under five. Participants reported seeing a median of five (IQR = 3–14, range = 0–70) children under five presenting with diarrhoea per week (**Figure 3**).

Almost all village doctors stocked medications (n = 370, 99.7%) and antibiotics (n = 370, 99.7%) at their practice location (**Table 2**). A small proportion (n = 67, 18.1%) reported receiving an honorarium for medication distribution. About a quarter administered intravenous fluids in their practice (n = 103, 27.8%).

**Table 2:** Antibiotics stocked and used to treat diarrhoea by village doctors practising in the Sitakunda Upazila (n = 371)

Antibiotic	Stocked, n (%)	Used to treat diarrhoea, n (%)
Metronidazole	369 (99.5)	324 (87.3)
Ciprofloxacin	367 (98.9)	349 (94.1)
Azithromycin	364 (98.1)	133 (35.8)
Nitazoxanide	325 (87.6)	281 (75.5)
Doxycycline	22 (5.9)	2 (0.5)
Ceftriaxone	17 (4.6)	9 (2.4)

When asked where they referred patients with paediatric diarrhoea, most reported they referred to the BITID (n = 181, 48.8%), a government-funded infectious disease hospital located in southern Sitakunda, or Sitakunda Upazila Health Complex (n = 178, 48.0%), a community hospital in northern Sitakunda. A small proportion reported referring cases to Chittagong Medical College Hospital (n = 12, 3.2%), a tertiary care hospital located in Chattogram, and none reported referring paediatric diarrhoea patients to private clinics.

### Potential for eCDST interventions

All village doctors (n = 371, 100%) had access to a phone, and the majority (n = 307, 82.7%) reported that their phones had internet access. Regarding cell phone coverage, the majority (n = 328, 88.4%) reported having a very good coverage at their practising location.

Most village doctors reported using their smartphones to support clinical decision-making (n = 291, 78.4%). When asked to report the tools they used, participants most commonly mentioned using Google (n = 176, 47.4%) and the Drug Information Management System (n = 74, 19.9%), an offline app developed by ITmedicus in Bangladesh to provide a comprehensive index of over 25 000 drugs [31].

## DISCUSSION

As of 2022, 60% of Bangladesh’s population resides in rural areas [32]. Informal health care providers, such as village doctors, serve as the primary source of care for almost half of this rural population [4,32]. The critical role in this context positions village doctors as important stakeholders who should be engaged to improve individual and population health. Because village doctors often sell antibiotics as part of their practice [16], understanding their treatment practices and characteristics can help inform interventions to reduce inappropriate use of antibiotics and improve antibiotic stewardship.

The location and characteristics of village doctors can offer insight into why many people seek care from village doctors instead of government clinics and hospitals. Their availability and accessibility demonstrate the reliance of rural populations on village doctors. Almost all village doctor practices reported being open seven days a week and taking house calls. Compared to government health care facilities, the greater accessibility of village doctors makes them an appealing and convenient option for those seeking immediate care [5]. The proximity of village doctors to households (median of 0.37 km) and the clustering of village doctor practices near major roadways (over half within 100 m) and urban areas further indicate the importance of accessibility and proximity as significant factors when choosing where to seek care. This overreliance on village doctors, who may not adhere to evidence-based guidelines, reflects broader systemic issues, including the lack of access to formal health care services in rural areas [33]. In many rural communities, formal health care facilities, such as government clinics and hospitals, are often scarce and may be located far from residential areas, making them inaccessible to much of the population [34]. Efforts to improve access to quality health care by improving the care village doctors provide, as well as increasing the accessibility of formal health care services, are critical to safeguarding public health outcomes, particularly in the context of antibiotic resistance.

Our findings suggest that antibiotic dispensing by village doctors in rural Bangladesh is prevalent and widespread. Almost all village doctors surveyed stocked antibiotics in their storefronts and reported commonly dispensing antibiotics for managing diarrhoea, with ciprofloxacin and metronidazole being the most frequently prescribed antibiotics. Given that diagnostic capabilities and facilities are limited in rural areas, providers often rely entirely upon empirical methods in managing diarrhoea, resulting in antibiotic use based solely on clinical suspicion. However, this method likely results in widespread misuse of antibiotics, as a recent study found that 63% of paediatric diarrhoea cases tested in rural Bangladesh were of a viral aetiology [35]. This discrepancy between village doctors’ clinical behaviours and national guidelines on antibiotic use, including Bangladesh’s National Drug Policy, which prohibits the sale and distribution of antibiotics without a prescription from a registered physician, underscores broader public health implications [36]. Current antibiotic dispensing practices highlight the urgent need for interventions to align clinical practices with evidence-based guidelines to mitigate the escalating threat of antimicrobial resistance. Additionally, the results emphasise the importance of strengthening diagnostic capabilities in rural health care settings to facilitate targeted and appropriate antibiotic prescribing [37].

Inappropriate use of antibiotics has a variety of detrimental consequences, both for individual and population health, making the high rate of antibiotic usage by village doctors a significant concern [19]. Village doctors’ poor knowledge of antibiotic resistance and stewardship significantly contributes to inappropriate antibiotic usage [38]. Studies have shown that informal health care providers have a limited understanding of antimicrobial resistance and the potential consequences of antibiotic overuse [8,39]. Additionally, incentives from pharmaceutical representatives, such as honorariums for medication distribution, may also influence their treatment practices [40]. Furthermore, patient demand for antibiotics and a perception of antibiotics as a cure for all ailments may further perpetuate high antibiotic dispensing rates [39,41]. High antibiotic use contributes to adverse drug reactions, treatment failure, and the development of antibiotic resistance. This is of particular concern, given the high prevalence of antibiotic-resistant pathogens in Bangladesh [17,42,43]. Throughout the country, there is widespread and developing resistance to both ciprofloxacin and metronidazole [17,44], the most commonly reported antibiotics prescribed for diarrhoea. Village doctors’ high rates of antibiotic use point to this group as an optimal target for antibiotic stewardship interventions. Additionally, understanding the landscape of antibiotic prescribing and distributing practices among village doctors in Bangladesh can provide insight into similar practices among informal health care providers in other countries, including India, Vietnam, and China, due to similarities in limited training, patient expectations, and antibiotic availability [45–47].

The availability and use of mobile phones suggest that eCDSTs could be feasible to improve antibiotic stewardship practices among village doctors. Almost all village doctors had access to internet-enabled phones, and most reported using resources on their phones for clinical decision-making. This familiarity with phone-based resources for clinical decision-making highlights the potential of eCDSTs as effective interventions that can further guide clinical practice and promote appropriate clinical management. The use of eCDSTs in low-resource settings offers the value of cost-effectiveness, sustainability, and accessibility [48]. eCDSTs can provide real-time guidance on proper antibiotic use, ensuring better adherence to treatment guidelines and promoting responsible antibiotic prescribing. Despite this, implementing eCDSTs poses several challenges. Many village doctors may lack sufficient digital literacy to effectively utilise these tools, requiring tailored training programmes to bridge this gap. Moreover, unreliable internet connectivity and inadequate infrastructure pose significant barriers, limiting access to eCDSTs and affecting their effectiveness. Furthermore, deep-rooted behavioural patterns among village doctors may resist the adoption of eCDSTs, highlighting the importance of targeted interventions to promote acceptance and integration into clinical practice [49]. Overcoming these challenges demands a comprehensive approach that addresses technical, educational, and cultural factors to ensure the successful implementation and utilisation of eCDSTs in low-resource settings [50].

In addition to the feasibility of eCDSTs to support changes in antibiotic dispensing practices, we also point to other opportunities to reduce inappropriate use of antibiotics. First, educational interventions and training programmes should be developed to enhance village doctors’ knowledge of appropriate antibiotic usage and the consequences of inappropriate antibiotic practices, as our results suggest that any intervention should account for the high variability in village doctors’ formal education level and medical-specific training. Tailoring educational interventions to accommodate diverse learning needs and levels of medical knowledge among village doctors is essential for maximising their effectiveness and ensuring the widespread adoption of best practices in antibiotic stewardship. Additionally, the fact that village doctors charged none to minimal fees for consultations suggests that the majority of their income comes from selling medications. Any intervention to reduce antibiotic dispensing will only be successful insofar as it provides an alternative source of income for village doctors.

Additionally, policy changes such as strengthening regulations on pharmaceutical marketing activities can help reduce the influence of incentives from pharmaceutical representatives [51]. Introducing measures to monitor and enforce compliance with these regulations can help prevent inappropriate marketing practices and mitigate their impact on antibiotic prescribing behaviour [52]. Importantly, integrating policy recommendations to address informal providers and creating frameworks for regulating informal health care providers is crucial. Countries such as India and Indonesia have implemented successful strategies in this regard. In India, the National Health Policy of 2017 recognises the need to integrate informal health care providers into the formal health care system through training, regulation, and accreditation programmes [53]. Similarly, Indonesia has established a community health worker programme to improve access to basic health care services in rural areas, while also implementing regulations to ensure the quality and safety of care provided by informal health care providers [54]. By drawing lessons from these successful initiatives, Bangladesh can develop comprehensive policies and regulatory frameworks that recognise the role of informal providers in the health care system while also protecting public health and promoting appropriate antibiotic use.

We should note some limitations of this study that may affect the interpretation of the results. The study's restriction to a specific geographic area within Bangladesh limits the extent to which its findings can be generalised to rural areas across the entire country. Rural regions may vary significantly in terms of demographics, health care infrastructure, and socio-economic factors, which could affect the behaviour and practices of village doctors. Therefore, conclusions drawn from this study may not apply to rural areas with different characteristics. Without the ability to verify the accuracy of participants' responses, particularly regarding their completion of the reported training, the reliability of the data are compromised. This introduces the potential for response bias, where participants may provide socially desirable or inaccurate information, leading them to misrepresent the scope of their clinical practice or their income. Consequently, the study's conclusions may be based on unreliable or incomplete data, impacting the robustness of its findings. The use of snowball sampling, while convenient, introduces inherent biases. It relies on participants' networks to identify additional participants, leading to a non-random sample that may not accurately represent the entire population of interest. In this case, there is a risk that village doctors who are not well-connected or are outliers within their communities may be underrepresented or excluded from the study. As a result, the findings may not fully capture the diversity of practices and perspectives among village doctors in the Sitakunda area.

Despite these limitations, the study offers a comprehensive understanding of the practices of village doctors in southeastern Bangladesh, specifically focussing on their antibiotic usage and prescribing practices for managing diarrhoeal illness. The study highlights the potential for eCDSTs to improve the practices of village doctors, directly informing interventions aimed at addressing irresponsible antibiotic usage and promoting antibiotic stewardship practices. These strengths collectively contribute to the significance of the study's findings in terms of understanding village doctors' practices, addressing issues related to antibiotic usage, and exploring paths for targeted interventions to enhance health care delivery in rural Bangladesh.

## CONCLUSIONS

Here we provided a comprehensive assessment of village doctors in the Sitakunda subdistrict of Bangladesh. The high density and accessibility of village doctor practices and the large number of paediatric diarrhoea patients seen each week by village doctors highlight the potential impact of working with this stakeholder group to improve antibiotic stewardship. The widespread use and availability of smartphone-based clinical support tools highlight eCDSTs as a potential solution for changing clinical practice, ultimately improving patient care, protecting public health, and reducing antibiotic resistance.

## Additional material


Online Supplementary Document
