# Supplementary material for: Identification, mapping, and self-reported practice patterns of village doctors in Sitakunda subdistrict, Bangladesh
Source: J Glob Health. 2024 Sep 13;14:04185. doi: 10.7189/jogh.14.04185 (PMC11393791; doi:10.7189/jogh.14.04185)
Supplement: Online Supplementary Document [file jogh-14-04185-s001.pdf]

Village Doctor Questionnaire

Question		Response
1.	Village Doctor ID:	
2.	Interview Date:	
3.	First Name:	
4.	Father's Name:	
5.	Age (years):	
6.	Gender	• Male<br>• Female
7.	Religion	• Islam<br>• Hindu<br>• Other (please specify)
8.	Where is your practice based (exact location)?	Location name (village/area):
9.	Practice location latitude:	
10.	Practice location longitude:	
11.	What type of practice do you have?	• Permanent building<br>• Roadside stand<br>• Home<br>• Pharmacy<br>• Door to door
a.	Do you own or rent the property where your practice is based?	• Own<br>• Rent
b.	Do you make house calls?	• Yes<br>• No
12.	How many people do you employ in your practice?	Number of employees:
13.	How many years have you been in practice as a village doctor?	Years:
a.	How many years have you been in practice as a village doctor in Sitakunda Upazila?	Years:
b.	How many years have you been in practice as a village doctor in your current location?	Years:
14.	What is the highest level of education you have obtained?	• Under class 5<br>• Class 6 – 8<br>• SSC completed<br>• HSC completed<br>• Graduation completed<br>• Post-graduation completed<br>• Other (please specify)

15.	What type of training did you undergo to be a village doctor? (Select all that apply.)	• Self-training<br>• MATS (Medical Assistant Training Course)<br>• LMAF (Local Medical Assistance and Family Planning)<br>• RMP (Rural medical partitional)<br>• DMS (Diploma in Medicine and Surgery)<br>• Bangladesh Nursing and Midwifery Council<br>• PCB (Pharmacy Council of Bangladesh)<br>• Medical Technologist<br>• Homeopathic Doctor<br>• BAMS (Bachelor of Ayurvedic Medicine & Surgery)<br>• Others (please specify)
a.	Did you complete any internship after your village doctor training?	• Yes<br>• No
b.	If yes, what was the internship's duration (months)?	Months:
16.	How many patients are seen at your practice/facility during an average week?	Number of patients:
a.	How many children under five years old do you see during an average week?	Number of patients:
b.	How many children under five years old do you see with diarrhea during an average week?	Number of patients:
17.	Do you have a clinical supervisor?	• Yes<br>• No
a.	If yes, what degree does that supervisor have?	• MBBS<br>• LMF<br>• Nursing<br>• Diploma in Medical Assistant<br>• Homeopathic doctor<br>• Ayurvedic<br>• None of the above
18.	What hours is your practice open to see patients?	• Sunday: XX-XX<br>• Monday: XX-XX<br>• Tuesday: XX-XX<br>• Wednesday: XX-XX<br>• Thursday: XX-XX<br>• Friday: XX-XX<br>• Saturday: XX-XX

a.	Do you perform on-call duties, such as treatment-related home visits?	• Yes<br>• No
19.	Do you work any other jobs other than as a village doctor?	• Yes<br>• No
20.	What fees do you typically charge as a village doctor?	Taka:
21.	What is your average monthly earning?	Taka/month:
22.	Do you receive any honorarium for prescribing/selling any specific drugs?	• Yes<br>• No
23.	Do you stock any medication at your location/shop/pharmacy?	• Yes<br>• No
24.	Which of the following antibiotics do you typically have in stock? (Select all that apply.)	• Metronidazole<br>• Azithromycin<br>• Doxycycline<br>• Ciprofloxacin<br>• Nitazoxanide<br>• Ceftriaxone<br>• Other (please specify)
a.	Which of the following antibiotics do you use to treat diarrhea? (Select all that apply.)	• Metronidazole<br>• Azithromycin<br>• Doxycycline<br>• Ciprofloxacin<br>• Nitazoxanide<br>• Ceftriaxone<br>• Other (please specify)
25.	Do you administer intravenous (IV) fluids at your location?	• Yes<br>• No
26.	If a child with diarrhea does not get better with your treatment, where do you send them? (Select all that apply.)	• Bangladesh Institute of Tropical and Infectious Diseases (BITID)<br>• Sitakunda Upazila Health Complex<br>• Chittagong Medical College<br>• Private Clinic<br>• Other (please specify)
27.	Do you have a phone?	• Yes<br>• No
a.	What is your mobile phone number?	Mobile phone number:
b.	What is the make and model of your phone?	Make and model:
c.	Is your phone a "smartphone" with access to the internet?	• Yes<br>• No
28.	Have you ever used your phone in any of the following ways to make decisions in your job?	• Using the internet to make a diagnosis<br>• Using the internet to decide on which drugs to use

		• Using the internet to decide on drug dosage<br>• Using an app on your phone that helps you with clinical decision-making<br>• Any other use of the internet to help in your clinical decision-making (please specify and describe)
a.	Who is your cell phone provider? (Select all that apply.)	• Grameenphone<br>• Banglalink<br>• Airtel<br>• Robi<br>• TeleTalk
b.	How good is your cell phone coverage in your shop?	• Not good at all<br>• Somewhat good<br>• Very good
